# Indolethylamine *N*-methyltransferase (INMT) is not essential for endogenous tryptamine-dependent methylation activity in rats

**DOI:** 10.1038/s41598-023-27538-y

**Published:** 2023-01-06

**Authors:** Nicolas G. Glynos, Lily Carter, Soo Jung Lee, Youngsoo Kim, Robert T. Kennedy, George A. Mashour, Michael M. Wang, Jimo Borjigin

**Affiliations:** 1 Department of Molecular and Integrative Physiology, University of Michigan, Ann Arbor, MI USA; 2 Michigan Psychedelic Center, University of Michigan, Ann Arbor, MI USA; 3 Department of Neurology, University of Michigan, Ann Arbor, MI USA; 4 Veterans Affairs Ann Arbor Healthcare System, Ann Arbor, MI USA; 5 Department of Chemistry, University of Michigan, Ann Arbor, MI USA; 6 Department of Anesthesiology, University of Michigan, Ann Arbor, MI USA; 7 Neuroscience Graduate Program, University of Michigan, Ann Arbor, MI USA

**Keywords:** Transferases, Biochemistry, Chemical biology, Neuroscience, Physiology

## Abstract

Indolethylamine *N*-methyltransferase (INMT) is a transmethylation enzyme that utilizes the methyl donor *S*-adenosyl-L-methionine to transfer methyl groups to amino groups of small molecule acceptor compounds. INMT is best known for its role in the biosynthesis of *N,N*-Dimethyltryptamine (DMT), a psychedelic compound found in mammalian brain and other tissues. In mammals, biosynthesis of DMT is thought to occur via the double methylation of tryptamine, where INMT first catalyzes the biosynthesis of *N*-methyltryptamine (NMT) and then DMT. However, it is unknown whether INMT is necessary for the biosynthesis of endogenous DMT. To test this, we generated a novel INMT-knockout rat model and studied tryptamine methylation using radiometric enzyme assays, thin-layer chromatography, and ultra-high-performance liquid chromatography tandem mass spectrometry. We also studied tryptamine methylation in recombinant rat, rabbit, and human INMT. We report that brain and lung tissues from both wild type and INMT-knockout rats show equal levels of tryptamine-dependent activity, but that the enzymatic products are neither NMT nor DMT. In addition, rat INMT was not sufficient for NMT or DMT biosynthesis. These results suggest an alternative enzymatic pathway for DMT biosynthesis in rats. This work motivates the investigation of novel pathways for endogenous DMT biosynthesis in mammals.

## Introduction

**Figure 1:**
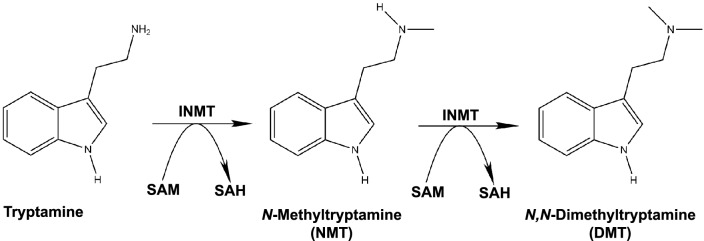
The Mammalian DMT biosynthetic pathway. The methylation of tryptamine to form *N*,*N*-Dimethyltryptamine is thought to occur via a two-step reaction catalyzed by the enzyme indolethylamine-*N*-methyltransferase (INMT), with *N*-methyltryptamine (NMT) as an intermediate. The co-factor *S*-adenosyl-L-methionine (SAM) serves as the methyl donor, with S-adenosyl-L-homocysteine (SAH) as a byproduct of this methyl transfer.

Indolethylamine *N*-Methyltransferase (INMT) is a transmethylation enzyme that catalyzes the transfer of methyl groups from the co-factor *S*-adenosyl-L-methionine (SAM) to amino groups of small molecule acceptor substrates^1^. The enzyme has a broad substrate specificity for several endogenous amines and other small molecules with tryptamine and *N*-methyltryptamine (NMT) being typically regarded as the enzyme’s primary substrates^2,3^. In a two-step reaction that utilizes SAM as methyl donor, INMT catalyzes the transfer of a methyl group to the amino side chain of tryptamine to first form NMT and then *N,N*-Dimethyltryptamine (DMT) (Fig. 1). This pathway was first described in 1961 by Axelrod^4^ who used a radiometric enzyme assay and thin-layer chromatography to demonstrate that rabbit lung extracts incubated with tryptamine and C^14^-SAM produced C^14^-NMT and C^14^-DMT. This study was one of the first to show that ‘psychotomimetic’ metabolites could be formed enzymatically from naturally occurring compounds.

INMT mRNA expression has been investigated in multiple mammalian species and has been reported in rabbit brain, liver, and lung^1^, several human tissues including heart, lung, liver, and adrenal gland^5^, as well as in brain, liver, lung, kidney, heart, and adrenal gland of rat^6^. In addition, INMT protein expression has been demonstrated in tissues from *Rhesus macaque* pineal gland, spinal cord, and retina^7^. Extracts from lung tissues—most notably from rabbit—have historically shown higher expression of INMT, and greater methylating activity toward tryptamine and NMT, relative to other tissues^1,8,9^. As a result, rabbit INMT was the first INMT to be cloned and characterized, with human INMT soon following^1,5^. When expressed in COS-1 cells, both recombinant proteins demonstrated sufficiency for tryptamine methylation with apparent K_m_ (mM) values of (mean ± SEM) 0.27 ± 0.05 and 2.92 ± 0.07 respectively^1,5^.

Although the endogenous roles of INMT are not fully understood, the enzyme is perhaps best known for its function in methylating endogenous tryptamine to form the doubly methylated psychedelic compound DMT^10^. DMT has structural similarity to the neurotransmitter serotonin (5-HT), it produces psychedelic effects when administered to humans^11^, and it occurs endogenously in a variety of plant species as well as mammalian tissues and bodily fluids^12^. The presence of endogenous DMT was first confirmed in humans in 1965 via blood and urine analysis^13^ and has subsequently been confirmed in blood, urine, and cerebrospinal fluid of humans with a variety of sophisticated analytical techniques, including gas chromatography (GC) and high-performance liquid chromatography (HPLC) coupled with mass spectrometry (MS) (see reference^14^ for a thorough review). However, in vivo sampling of DMT in the brain has been restricted to rodent studies, with rats being the most widely studied species for this purpose. Several reports have confirmed the presence of endogenous DMT in rat brain and other tissues. Saavedra and Axelrod performed intracisternal injections of C^14^-tryptamine in rats and demonstrated whole brain recovery of C^14^-NMT and C^14^-DMT via thin-layer chromatography with multiple solvent systems^15^. Beaton and Morris detected and quantified DMT from whole brain extracts of untreated rats at varying developmental stages using GC-MS^16^. Kärkkäinen et al. pre-treated rats with a monoamine oxidase inhibitor (MAOI) and found DMT present in kidney, lung, liver, and brain tissues using HPLC-tandem mass spectrometry (MS/MS)^17^. Barker et al. analyzed perfusate samples collected via microdialysis in occipital cortex of freely behaving untreated rats and confirmed the presence of DMT using LC–MS/MS^18^. Finally, Dean et al. used HPLC with fluorescence detection to quantify DMT in perfusate samples collected from occipital cortex of freely behaving untreated rats and showed that endogenous DMT levels ranged from 0.05 to 2.2 nM^6^.

There are certain aspects of INMT biochemistry that have precluded a clear understanding of its functions and role in tryptamine methylation. This is in part due to the fact that many early studies examining INMT function relied on ex vivo assays utilizing crude or partially purified protein extracts. These techniques are useful for understanding broad enzymatic activity of tissues but are insufficient for characterizing the activity of a single enzyme. One indicator of this limitation is the reported promiscuity of enzymatic activity from protein extracts. Indeed, multiple studies that utilized extracts from various tissue sources reported that enzymes from these tissues interact with up to three dozen distinct substrates including endogenous compounds and xenobiotics^2,3,19^. These findings were often broadly characterized as resulting from “INMT activity,” but the methodologies were insufficient for that characterization. Another indicator of the limitations of these techniques is that protein extracts appear to exhibit inconsistent levels of activity and affinity toward substrates when assay conditions are varied, or when sourced from different tissues within the same species^10^. For example, using enzymes from crude rabbit lung extracts, Axelrod identified 5-HT as the preferred substrate, with tryptamine and NMT showing 81% and 39% of the activity of 5-HT, respectively^3^. However, other studies using enzyme from the same source found NMT to be the preferred substrate with a K_m_ value of 50 µM, followed by tryptamine with a K_m_ of 330 µM^8,9,20^. Enzyme activity assayed from crude chick brain extract showed 5-HT to be the preferred substrate, with tryptamine and NMT showing 60% and 47% relative activity^21^, respectively, and a study with human brain extracts showed tryptamine to be the preferred substrate (111%), compared to 5-HT (100%), and NMT (55%)^2^. Finally, enzyme activity from crude rat brain extracts showed additional inconsistencies, where tryptamine was the preferred substrate (K_m_ = 27.8 µM) followed by NMT (K_m_ = 36.8 µM)^22^.

A more direct approach to study the activity of a specific enzyme of interest is the utilization of purified recombinant proteins. Thompson and colleagues were the first to take this approach, as they cloned, expressed, and characterized both rabbit and human INMT, confirming that both of these enzymes do indeed methylate tryptamine^1,5^. However, despite the fact that rats have served as the model species for endogenous DMT detection in the brain, no studies to date have attempted to characterize the activity of recombinant rat INMT or investigate rat INMT using modern transgenic approaches.

To better understand the role that INMT plays in the biosynthesis of endogenous DMT, we utilized CRISPR/Cas9 to develop a novel INMT deficient rat model. Rats were chosen because they have been the model species for in vivo detection of DMT to date. Because a principal function of INMT has long been assumed to be the methylation of tryptamine to form NMT and DMT, we hypothesized that tissues from INMT deficient rats would lack these functions. To test this hypothesis, we used littermate pairs from INMT+/− crosses to compare tryptamine-methylating activity in brain and lung tissues between INMT +/+ (WT) and INMT −/− (KO) rats. Prior to this study, investigation of recombinant INMT had been limited to only rabbit and human INMTs, so we also compared the tryptamine-methylating activity of recombinant rat, rabbit, and human INMT expressed in *E. coli*. To study enzyme activity, including the identification and quantification of reaction products of enzyme assays, we used a battery of experimental techniques and analytical tools including radiometric enzyme assays with scintillation counting, thin-layer chromatography with phosphor imaging, and ultra-high-performance liquid chromatography tandem mass spectrometry (uHPLC-MS/MS).

## Results

### The deletion of INMT does not affect tryptamine-dependent enzyme activity in brain or lung tissues of rats

**Figure 2:**
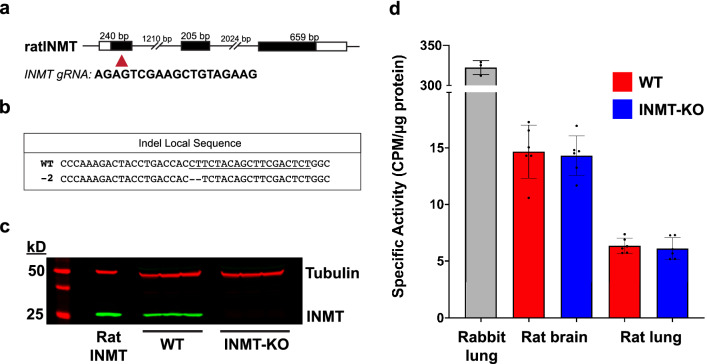
Enzymatic methylation of tryptamine does not differ between WT and INMT-KO rats (**a**) The gene structure of rat INMT with exons represented in black boxes. The location and sequence of the INMT guide RNA (gRNA) is shown with a red triangle. (**b**) The 2-base pair deletion responsible for generating the KO is shown below the wild type (WT) sequence with the location of the gRNA underlined. (**c**) Western blot showing the presence of INMT (green bands) in WT, but not KO rat lung tissues. The original image including all lanes in the molecular weight marker is included as Supplementary Figure S1. (**d**) Radiometric enzyme assays with tissue extracts from rabbit lung, as well as rat brain and rat lung from both WT and INMT-KO rats. Each point represents the average of 3 triplicate experiments from an individual animal for rabbits (n = 3), and rats (n = 6) for each genotype. Plots show averages with error bars indicating standard deviations. CPM: counts per minute. Rabbit lung tissue extracts (n = 3) showed tryptamine-dependent activity > 20-fold higher than rat tissues (average specific activity [SA] = 322.2 ± 8.81, 95% confidence interval [CI] of mean = 300.3–344.0). Tryptamine-dependent activity did not differ between WT and KO rats in brain (t = 0.30, mean difference = − 0.36, 95% CI = − 3.05–2.33, *p* = 0.77) or lung tissues (t = 0.52, mean difference = − 0.25, 95% CI = − 1.33–0.84, *p* = 0.62). In brain, average SA was 14.68 ± 2.34, 95% CI of mean = 12.22–17.13 in WT, and 14.32 ± 1.76, 95% CI of mean = 12.47–16.16 in KO. In lung, average SA was 6.36 ± 0.66, 95% CI of mean = 5.66–7.05 in WT, and 6.11 ± 0.96, 95% CI of mean = 5.10–7.12 in KO. Tryptamine-dependent activity was significantly higher in brain tissues relative to lung (*t* = 8.38, mean difference = 8.32, 95% CI = 5.87–10.77, p = 0.0002 for WT and *t* = 10.04, mean difference = 8.21, 95% CI = 6.31–10.11, *p* < 0.0001 for KO).

Using CRISPR/Cas9, a 2-nucleotide deletion was introduced into exon 1 of the rat INMT gene (Fig. 2a), resulting in a frameshift mutation (Fig. 2b) and absence of INMT protein expression in the rat (Fig. 2c). Presence or absence of INMT expression in rats was confirmed with PCR products from tail snips, and Western blots using lung tissue homogenates. The deletion of INMT did not result in overt behavioral or physiological abnormalities, and rats were reproductively viable. To determine the impact of INMT deletion on tryptamine-dependent enzyme activity, we conducted radiometric enzyme assays using brain and lung tissues from both WT and KO rats; rabbit lung tissue extracts were used as positive control (Fig. 2d). These highly sensitive assays rely on the transfer of a radiolabeled (C^14^) methyl group from C^14^-SAM to a substrate, where activity is measured via liquid scintillation counting. The data indicate that deletion of rat INMT had no detectable impact on the ability of brain or lung extracts to methylate tryptamine.

### Rat brain and lung enzymes methylate tryptamine to produce unknown reaction products

**Figure 3:**
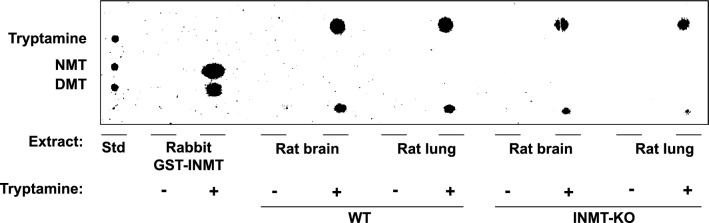
Brain and lung tissues from WT and KO rats produce products that are not isographic with NMT or DMT. Phosphor imaging scan of radiometric enzyme assay products using extracts from brain and lung tissues of WT and KO rats following separation on silica gel plates using a mobile phase of *N*-butanol:acetic acid:water (12:3:5). A standard mixture (Std) containing tryptamine, NMT and DMT were clearly separated using these chromatography methods (left most lane). Uncropped and unprocessed phosphor imaging scan of the graph is included as Supplementary Figure S2.

To characterize tryptamine methylation activity from brain and lung tissues of WT and KO rats, thin-layer chromatography was first used to assess the reaction products, and uHPLC-MS/MS was implemented to provide additional validation and quantification. With thin-layer chromatography, tryptamine, NMT and DMT standards produced retardation factor (R_F_) values of 0.65, 0.55, and 0.47, respectively (Fig. 3, left). Extracts from rabbit glutathione-*S*-transferase-INMT fusion proteins (GST-INMT) expressed in *E. coli* (positive control) produced two distinct spots that were isographic with both NMT (R_F_ = 0.53) and DMT (R_F_ = 0.46). Rat tissues, including brain and lung of both WT and KO rats, did not produce spots isographic with either NMT or DMT, but did produce two distinctive spots with R_F_ values of 0.67 (top), and 0.37 (bottom). All conditions where tryptamine was omitted showed no detectable methylating activity.

For uHPLC-MS/MS analysis of reaction products from tissue extract assays (Table 1), tryptamine was omitted from the corresponding tissue for each condition to serve as background control for NMT levels.

**Table 1:** uHPLC-MS/MS confirmation of enzymatic activity of brain and lung tissues of WT and KO rats. For rats, values shown are averages of duplicates from each of n = 6 rats per genotype ± standard deviation. For rabbit lung, values shown are averages of a duplicate ± standard deviation. TA = tryptamine, NF = peak not found.

Extracts	TA	NMT (nM)	DMT (nM)
WT rat brain	−	0.18 ± 0.09	NF
WT rat brain	+	0.15 ± 0.09	NF
KO rat brain	−	0.29 ± 0.13	NF
KO rat brain	+	0.11 ± 0.06	NF
WT rat lung	−	0.44 ± 0.22	NF
WT rat lung	+	0.6 ± 0.67	NF
KO rat lung	−	0.68 ± 0.23	NF
KO rat lung	+	0.44 ± 0.26	NF
Rabbit lung	−	0.08 ± 0.05	NF
Rabbit lung	+	24.2 ± 13.2	0.57 ± 0.18

Rabbit lung tissue extracts were used as a positive control, and results indicate that these extracts produced NMT at a level of nearly 300-fold that of the background level, and DMT at a level of (mean ± standard deviation) 0.57 ± 0.18 nM. This activity was dependent on the addition of tryptamine to the reaction mixture. Brain and lung tissues from both WT and KO rats, however, did not show tryptamine dependent production of NMT or DMT. These results are consistent with thin-layer chromatography findings, and confirm that under these assay conditions, NMT and DMT are not produced by enzymes present in brain or lung tissues of rat.

### Rat INMT is not sufficient for tryptamine methylation

**Figure 4:**
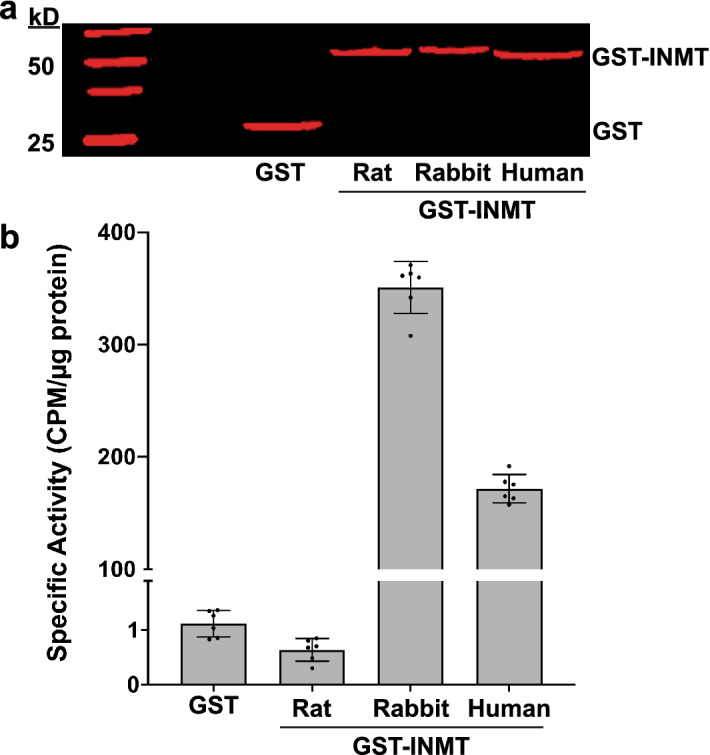
Rat INMT is not sufficient for tryptamine methylation. (**a**) Western blot showing expression of GST-INMT fusion proteins of rat, rabbit and human INMTs in E. coli. The control lane is showing expression of an empty vector GST, where INMT is absent. Uncropped image of the western blot is included as Supplementary Figure S3*.* (**b**) Radiometric enzyme assays with extracts from E. coli expressing GST-fusion recombinant rat, rabbit, and human INMT proteins. Plots show the average of 6 individual experiments per condition with error bars indicating standard deviations. CPM = counts per minute. The assay revealed tryptamine-dependent activity of both rabbit INMT (average SA = 350.9 ± 23.08, 95% CI of mean = 326.7–375.1) and human INMT (average SA = 171.8 ± 12.38, 95% CI of mean = 158.8–184.8). Activity was significantly higher in rabbit INMT relative to human INMT (*t* = 16.75, mean difference = 179.1, 95% CI = 154.3–204, p < 0.0001). The activity of rat INMT (average SA = 0.64 ± 0.21, 95% CI of mean = 0.42–0.85) was significantly lower (*t* = 3.68, mean difference = − 0.48, 95% CI = − 0.77 to − 0.19, *p* = 0.004) than empty vector GST extracts (average SA = 1.12 ± 0.24, 95% CI of mean = 0.86–1.37).

Extracts from *E.coli* cells expressing recombinant rat, rabbit, and human GST-INMT fusion proteins were used for the radiometric quantification of tryptamine-dependent activity of each of these enzymes (Fig. 4). These data demonstrate robust tryptamine-dependent activity of both rabbit and human INMT and indicate that rat INMT is not sufficient for tryptamine methylation.

To rule out the possibility that an inhibitory factor within the *E.coli* extracts might be contributing to the lack of enzyme activity from rat INMT, we performed identical experiments using Flag-tagged recombinant rat, rabbit and human INMT proteins expressed in human embryonic kidney (HEK) 293 cells (Supplementary Figure S4). To determine whether rat INMT has the capacity to methylate NMT, the assays were conducted using GST-INMT fusion proteins and NMT as a substrate, and similar results were observed (Supplementary Figure S5). Rat INMT did not show enzymatic activity toward NMT, but rabbit and human INMTs were both highly active.

### Rat INMT is not capable of methylating tryptamine to form NMT and DMT

**Figure 5:**
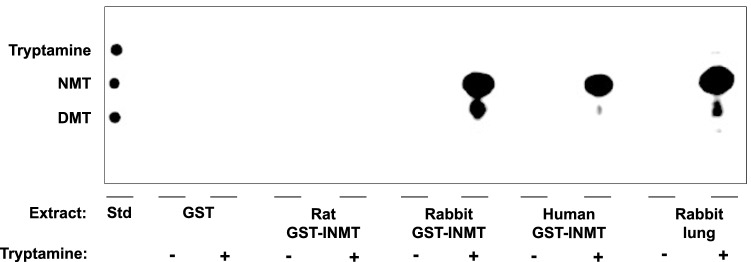
Rabbit and human, but not rat INMT methylate tryptamine to produce NMT and DMT. Phosphor imaging scan of radiometric enzyme assay products using recombinant GST-INMT fusion proteins following separation on silica gel plates using a mobile phase of N-butanol:acetic acid:water (12:3:5). A standard (Std) mixture containing tryptamine, NMT and DMT were clearly separated using these chromatography methods (left most lane). The standards showed respective R_F_ values of 0.58, 0.49, and 0.39. Rabbit INMT, human INMT, and rabbit lung each showed two spots isographic with both NMT and DMT. For rabbit INMT, NMT R_F_ = 0.48 DMT R_F_ = 0.41. For human INMT, NMT R_F_ = 0 48 and DMT R_F_ = 0.41. For rabbit lung (positive control), NMT R_F_ = 0.50 and DMT R_F_ = 0.41. Neither empty vector GST preparations nor rat GST-INMT showed detectable products, which indicates a lack of methylation activity. Uncropped, unprocessed phosphor imaging scan of the same graph is included as Supplementary Figure S6.

Thin-layer chromatography coupled with phosphor imaging was used to identify the tryptamine-dependent reaction products of radiometric recombinant enzyme assays described above. Pure solutions of unlabeled tryptamine, NMT and DMT standards were clearly separated using this solvent system (Fig. 5, left).

Recombinant INMT proteins from rabbit and human showed robust tryptamine-dependent methylation activity, while rat recombinant INMT protein had no detectable activity toward tryptamine.

When NMT was used in place of tryptamine as a substrate, similar results were observed (Supplementary Figure S7): rat INMT did not produce methylated products, but rabbit and human INMTs produced DMT as indicated by the presence of spots that were isographic with a DMT reference standard. Additionally, all conditions in which tryptamine (Fig. 5) or NMT (Supplementary Figure S7) was omitted showed no detectable methylation, confirming that the reactions are substrate dependent.

Validation of these findings was carried out using uHPLC-MS/MS. For recombinant INMT studies, empty vector GST-transformed *E. coli* extracts incubated with tryptamine and SAM served as background controls, and the NMT value from those assays was defined as background. These assays showed that tryptamine-dependent production of NMT and DMT occurred only with rabbit and human INMT, where rabbit INMT showed NMT production > 50-fold higher than the background controls, and human INMT > 60-fold higher (Table 2). In contrast to rabbit and human INMT, rat INMT did not show detectable tryptamine-dependent production of NMT or DMT. The concentrations of enzymatically produced DMT resulting from these assays were (mean ± standard deviation) 0.23 ± 0.03 and 0.12 ± 0.06 for rabbit and human INMT respectively.

**Table 2:** uHPLC-MS/MS confirmation of enzymatic activity of recombinant rat, rabbit, and human INMTs. Values shown are averages of triplicates for each condition ± standard deviation. TA = tryptamine, NF = peak not found.

Extracts	TA	NMT (nM)	DMT (nM)
GST	+	0.37 ± 0.48	NF
Rat GST-INMT	−	0.39 ± 0.07	NF
Rat GST-INMT	+	0.14 ± 0.07	NF
Rabbit GST-INMT	−	0.13 ± 0.03	NF
Rabbit GST-INMT	+	19.86 ± 1.86	0.23 ± 0.03
Human GST-INMT	−	0.26 ± 0.04	NF
Human GST-INMT	+	22.59 ± 6.72	0.12 ± 0.06

When NMT was used in place of tryptamine as a substrate, the production of DMT was confirmed only from rabbit and human INMTs, and rat INMT was inactive (Supplementary Table S1). For all conditions, the omission of tryptamine or NMT substrates resulted in undetectable levels of NMT and DMT products. In addition to providing precise quantification of analytes, these results correspond closely with those from radiometric enzyme assays and provide validation of thin-layer chromatography results.

## Discussion

We report for the first time the generation of a novel transgenic INMT-KO rat model, as well as comprehensive multi-species study of INMT in relation to tryptamine-dependent activity and the biosynthesis of NMT and DMT. Additionally, this study is the first to investigate the tryptamine-dependent activity of recombinant rat INMT, which is relevant for the field because rat is currently the model species for in vivo DMT detection in the brain. The combination of traditional techniques (radiometric enzyme assays and thin-layer chromatography) and modern techniques (CRISPR/Cas9 developed INMT-KO rats, multi species recombinant INMT comparisons, and uHPLC-MS/MS), results in an informative characterization of INMT function, tryptamine methylation, and the biosynthesis of endogenous DMT.

The primary finding of this investigation is that INMT is not necessary for tryptamine methylation and biosynthesis of NMT and DMT in brain or lung tissues of rats. Using a highly sensitive radiometric enzyme assay, we show that extracts from brain and lung tissues of both WT and KO rats display equal levels of tryptamine-dependent activity under these assay conditions. When examining recombinant INMT expressed in *E. coli* cells, we found that rat INMT does not show tryptamine-dependent activity, while rabbit and human INMT are both highly active. This is likely due to the low degree of sequence homology of INMT between species. Although the sequences of rabbit and human INMT are nearly identical (89%), rat INMT is only 57% homologous with either. Together, these results demonstrate that rat tissues possess an enzyme that shows tryptamine-dependent methylation activity, but that the enzyme responsible is not INMT.

Thin-layer chromatography coupled with phosphor imaging revealed that the methylated products from rabbit lung tissues, as well as rabbit and human recombinant INMT were isographic with NMT and DMT. Rat INMT, however, did not result in the biosynthesis of any detectable methylated products, indicating that rat INMT does not catalyze the methylation of tryptamine. Interestingly, concurrent with radiometric assay results, tissue extracts from brain and lung of WT and KO rats showed equal tryptamine methylation activity, producing two distinctive spots when subjected to thin-layer chromatography. However, neither of these spots were isographic with NMT or DMT. These results support the conclusion that the identity of the enzyme that methylates tryptamine is not INMT, and further show that the products of that unidentified enzyme reaction are neither NMT nor DMT. Finally, uHPLC-MS/MS experiments replicated and validated thin-layer chromatography results—rabbit lung, as well as recombinant rabbit and human (but not rat) INMT can produce NMT and DMT under these assay conditions, and tissue extracts from brain and lung of WT and KO rats do not produce NMT or DMT. Taken together, in conjunction with the fact that DMT has been independently detected in the rat brain by multiple groups^6,15–18^, these results suggest that an alternative biosynthetic pathway for NMT and DMT is present in brain and lung tissues of rats. It is therefore likely that a distinctive mammalian *N*-methyltransferase exists that can methylate tryptamine to produce NMT and DMT.

There is biochemical evidence to support the possible existence of multiple enzymes that modify tryptamine. The pioneering work of Axelrod in 1961^4^ utilized crude extracts taken from rabbit lung tissues to assay tryptamine methylation activity, a relatively common practice in enzyme assay methodology. The vast majority of INMT studies over the subsequent three decades utilized similar strategies with crude or partially purified tissue extracts as protein sources for so-called INMT assays. Indeed, as more sophisticated protein purification methods became available, more recent studies made note of the likely existence of distinctive proteins with tryptamine-methylating activity. Using partially purified rabbit lung extracts, Porta et al. described two distinctive INMT-like tryptamine methylating proteins with similar enzyme kinetics, but distinctive isoelectric points and pH optima^23^. Additionally, Ansher and Jakoby identified what they labeled as “Methyltransferase A” and “Methyltransferase B” from purified rabbit liver extracts^19^. These two forms of INMT showed tryptamine methylating activity and had the same molecular weight but differed in their isoelectric points and substate interactions. Our results motivate similar studies in tissues of rat and human to determine if alternative tryptamine methylating enzymes are present.

It is well known that the behavior of individual enzymes under in vitro conditions is highly dependent upon a number of experimental variables including pH, temperature, ionic strength, concentration of assay components, buffer composition, and interactions of various co-factors or components not directly involved in the reaction^24^. It is therefore reasonable to conclude that under the current in vitro conditions, the methylation of tryptamine to form NMT and DMT in crude extracts from rat brain and lung is not possible. Instead, these conditions favored the activity of an alternative enzyme, which contributed to the production of the unidentified products shown in Fig. 3. Several studies have utilized in vivo approaches to demonstrate the presence of endogenous DMT in the rat brain^6,15,17,18,25^, highlighting the importance of in vivo physiological conditions for the methylation of tryptamine in rat brain tissues. We therefore speculate that an exploration of alternative assay conditions or a more thorough enzyme screening would result in the detectable production of NMT and DMT in rat tissues. These studies will be essential to better understand the function, regulation, and biosynthetic pathways(s) of endogenous DMT.

The identity of the products produced from brain and lung extracts of WT and KO rat tissues are currently unknown, but likely candidates for these compounds may be the β-carbolines and their associated alkaloids. β-carbolines are an abundant class of compounds that serve as MAOIs, and are characterized by their tricyclic, pyridine-fused indole framework. To date, more than 500 tricyclic β-carboline alkaloid monomers have been isolated from natural sources^26^. Interestingly, tryptamine has long been known to serve as a reactant in the biosynthesis of various β-carbolines via the Pictet-Spengler reaction, a chemical reaction resulting in condensation of tryptamine and subsequent ring closure^27^. This reaction can occur non-enzymatically, or enzymatically via the enzyme strictosidine synthase^28^. Additionally, multiple β-carboline alkaloids have been shown to be present within brain tissues of mammals including rats^29^, and other studies have reported on the possible formation of β-carboline alkaloids using similar assays to the current study^30,31^. With this mechanism in mind, it is therefore reasonable to hypothesize that the identities of the products resulting from incubation of rat brain and lung tissues with tryptamine may indeed be β-carboline alkaloids. Regardless of the identities of the unknown reaction products, the enzymatic production of these tryptamine-derived compounds is clearly independent of INMT, indicating the existence of an alternative enzyme that modifies tryptamine in mammals. An investigation of these products and their respective biosynthetic pathways will likely lead to a more thorough understanding of tryptamine metabolites in mammalian physiology. The availability of INMT-KO rats should facilitate further understanding of the endogenous function of this enzyme in rats.

In conclusion, our results confirm that rabbit INMT, human INMT, and rabbit lung tissue extracts are all capable of methylating tryptamine to form both NMT and DMT. However, rat INMT and tissue extracts from rat brain and rat lung do not produce NMT or DMT under the currently studied conditions. Rather, rat tissues interact with tryptamine to produce alternative products, and INMT is not necessary for this reaction. Since the in vivo presence of DMT in the rat brain is well established, these results suggest the potential existence of an alternative biosynthetic pathway for DMT in mammalian physiology.

## Methods

### Animal care and transgenic rats

All animals were housed in a 12:12 light:dark cycle beginning at 7 am and were provided ad libitum access to food and water. The study was approved by the Animal Care and Use Committee at the University of Michigan in Ann Arbor, and all methods were performed in accordance with the relevant guidelines and recommendations of that committee. INMT-KO rats were generated in an F344 background using CRISPR/Cas9 at The Transgenic Animal Model Core at the University of Michigan via a 2-base pair deletion in exon 1 of the INMT coding sequence. At postnatal day 21, rat pups were weaned and 1 mm sections of tails were collected into sterile tubes for genotyping using a standard DNA extraction procedure, and rats were identified as either WT (INMT + / +), KO (INMT −/−), or heterozygous (INMT + /−). For enzyme assays comparing WT vs. KO rats, heterozygous rats were bred to produce litters containing both genotypes, and littermates were used for INMT assay comparisons between WT and KO rats. Exclusion criteria for rats were defined a priori and included (1) a heterozygous genotype, and (2) any obvious behavioral or physiological abnormality. Therefore, only WT and KO rats were used in the study, and all rats were healthy at the time of sacrifice. All procedures were in compliance with recommended ARRIVE guidelines.

### Generation of GST-INMT and expression in *E. coli*

Rat (NM_001109022.1), rabbit (NM_001082043.1), and human (NM_006774.5) INMTs were generated by PCR using the following primer sequences: the human sense and anti-sense primers were 5′-AAAAGGATCCATGAAGGGTGGCTTCACTGGG-3′, and 5′-AAAAGAATTCTCAGGGCCCAGGCTTCTTGCG-3′, respectively. The rabbit sense and anti-sense primers were 5′-AAAAGGATCCATGGAGGGCGGCTTCACGGG-3′ and 5′-AAAAGAATTCTCAGGACCCCGGCTTCTTGC-3′ respectively. The rat sense and anti-sense primers were 5′-AAAAGGATCCATGGCAGGCAAGGTATACATG-3′ and 5'-AAAAGAATTCTCAGGCACTGGGACCCTTTCG-3′ respectively. Each of the clones were engineered into pGEX-2TK bacterial expression vectors using BamH I and EcoR I restriction enzymes. These vectors contained glutathione-*S*-transferase (GST) genes which serve to increase the solubility and expression of recombinant INMTs. Clones were transformed into BL21 (DE3) (Sigma Aldrich #CMC0015) for expression in *E. coli.* Colonies were inoculated into 25 mL LB ampicillin at 37 °C for 15–18 h. The cells were then harvested, pelleted, the supernatant was discarded, and the cells were frozen at − 20 °C prior to cell lysis and dialysis.

### Preparation of protein extracts and western blot analysis

For brain and lung extracts, rats (10 weeks old) were euthanized via CO_2_ inhalation and decapitated. Whole brain and lungs were quickly extracted, diced, and immediately frozen on dry ice in microcentrifuge tubes. Frozen rabbit lung tissues were obtained from Pel-Freez Biologicals. Frozen tissues (rat brain or lung, or rabbit lung) and frozen GST-INMT pellets were resuspended in 5 volumes of 10 mM sodium phosphate buffer (pH 7.9) and homogenized with three bursts at 6000 rpm. Homogenates were centrifuged at 100,000 g for 60 min at 4 °C. Supernatants were dialyzed overnight at 4 °C with 100 volumes of sodium phosphate buffer with 3 buffer changes every 4 h, using 12 kDa molecular weight cutoff Pur-A-Lyzer Maxi Dialysis Kits (Sigma Aldrich). Protein concentrations of dialyzed supernatants were determined via Bradford Assay (Bio-Rad), and remaining fractions were aliquoted and stored at -80 °C for enzyme assays. To confirm INMT expression, all tissue and GST-INMT extracts were analyzed via Western blot. 30 µg of each protein was denatured in phosphate-buffered saline containing β-mercaptoethanol and boiled at 100 °C for 5 min. Samples were separated on standard SDS–polyacrylamide gels and electroblotted to nitrocellulose. Western blot analysis was performed with rat INMT (rabbit polyclonal custom ordered from GenScript), tubulin (mouse monoclonal from Developmental Studies Hybridoma Bank), and GST (mouse monoclonal from Fisher) antibodies, followed by incubation with infrared fluorophore-labeled secondary antibodies against rabbit and mouse (Li-COR). Bands were detected using a Li-COR Odyssey infrared scanner. Resulting western blot images (Figs. 2 and 4) are presented as cropped versions of the unprocessed raw files. See supplemental materials for uncropped images.

### Enzyme assays

Protein sources for enzyme assays included tissue extracts (rabbit lung, rat brain, rat lung) and recombinant GST-INMTs (rat, rabbit and human). Enzyme assays were conducted in 1.5 mL microcentrifuge tubes and contained approximately 50 µg protein (stock protein concentrations ranged from 4.1 to 6.9 mg/mL for rat brain, 10.6–14.6 mg/mL for rat and rabbit lung, and 0.75–1.05 mg/mL for recombinant GST-INMTs). Radiometric enzyme assays utilized 4 mM tryptamine (Sigma Aldrich) or NMT (Cayman Chemical) along with 2.3 nmol C^14^-SAM (specific activity 52.6 mCi/mmol, PerkinElmer Inc.) in a final reaction volume of 150 µL with sodium phosphate buffer. Assay components were combined and incubated at 37 °C for 60 min, and reactions were stopped by adding 1 mL ice cold 0.5 M borate buffer (pH 9.5). To extract reaction products, the reaction mixtures were transferred to 15 mL Falcon tubes with 6 mL of a 97:3 toluene:isoamyl alcohol (T:IAA) solution and tubes were vortexed for three bursts of 5 s each and centrifuged at 1,650 g for 3 min. For quantification, 4 mL of the T:IAA organic layer was added to scintillation vials containing 5 mL scintillation cocktail (Ultima Gold; PerkinElmer Inc.), and radioactivity was counted for 1 min on a Beckman LS Scintillation Counter. Tubes with substrate (tryptamine) omitted served as background controls. Specific activity (SA) was calculated by subtracting the average CPM of 3 background controls for each condition and dividing the resulting CPM value by the amount of protein (µg) used. Results are presented as averages of triplicate experiments in both brain and lung tissues for *n* = 6 rats per genotype, and as 6 replicate experiments for each GST-INMT condition. Non-radioactive assays were carried out for analysis via uHPLC-MS/MS analysis. These assays utilized 1 mM tryptamine or NMT with 33 µM SAM in a final reaction volume of 150 µL with sodium phosphate buffer. Assay components were combined and incubated at 37 °C for 60 min. Tubes with substrate omitted served as background controls. Reaction products were immediately extracted by adding 1 mL T:IAA and vortexed. 950 µL of the organic phase was transferred to a new 1.5 mL tube, and the organic solvent was evaporated on a bench top VacuFuge™ concentrator (Eppendorf) at room temperature for 2 h. The resulting products were resuspended in 1 mL 30% methanol, vortexed for 3 min, and analyzed via uHPLC-MS/MS (see below for “Methods”). Samples that had NMT as a substrate were further diluted tenfold in 30% methanol. Assays with tissue extracts were run in duplicate for each rat (*n* = 6 per genotype), and assays with GST-INMT extracts were run in triplicate for each condition, with empty vector GST transformations serving as background controls. Sample identities were blinded by L.C. for uHPLC-MS/MS analysis conducted by N.G.G.

### Thin-layer chromatography and phosphor imaging

For product analysis, INMT assays were repeated for all tissues and recombinant GST-INMTs using the methods described above for radiometric enzyme assays. Reactions were prepared in quadruplicate with or without tryptamine in 1.5 mL microcentrifuge tubes. For product extraction, 1 mL T:IAA was added to the reaction tubes immediately following the assay. The tubes were vortexed three times at 5 s intervals and centrifuged at 13,000 rpm for 3 min. The supernatant was then transferred to clean microcentrifuge tubes and evaporated at 50 °C overnight in Eppendorf Thermomixer. The resulting products from four replicates for each condition were pooled together using 100 µL T:IAA. Prior to spotting, silica gel thin-layer chromatography plates were activated in an oven at 90 °C for 30 min, and a glass thin-layer chromatography development tank was prefilled with 50 mL mobile phase consisting of *N*-Butanol, acetic acid, and water (12:3:5). Plates were spotted with a mixture of standards including 0.5 µg each of tryptamine, NMT and DMT in T:IAA along with 50 µL of pooled enzyme assay reaction products, all spotted 1.0 cm apart. The plate was placed in the developing tank for approximately 2 h, then removed and allowed to air dry for 1 h. Tryptamine, NMT and DMT standards (viewable under ultraviolet light), as well as the product origins and solvent fronts were over-spotted with a diluted mixture of C^14^-SAM to allow for these locations to be visible with phosphor imaging. For development, the plate was wrapped in plastic wrap and stored in a phosphor screen (Molecular Dynamics) for 72 h. Imaging of the screen was done using an Amersham Typhoon Phosphorimager with photomultiplier tube sensitivity at 1,000v, normal scan speed, and pixel size of 100 µm. Resulting images (Figs. 3 and 5) were cropped to exclude spotting lines and solvent fronts, and globally modified with Amersham Typhoon imaging software to enhance visibility of reaction products. Raw, uncropped and unprocessed images are provided in the supplementary materials. Retardation factor (R_F_) values were determined by dividing the migration distance of the solute by the migration distance of the solvent front.

### Ultra-high-performance liquid chromatography tandem mass spectrometry

Although radiometric assays are highly sensitive in their ability to detect extremely low levels of enzyme activity, they do not provide information on identities of the reaction products. Thin-layer chromatography is useful for visualization of a range of reaction products, but the technique often lacks resolution. uHPLC-MS/MS, however, provides both a high level of sensitivity and resolution in analyte identification and represents a gold standard for analyte separation, detection, and quantification. Additionally, our uHPLC-MS/MS method utilizes multiple techniques to definitively confirm analyte identification, including retention time via uHPLC, triple quadrupole multiple reaction monitoring of both precursor and product ions, and spiked deuterated internal reference standards for NMT and DMT.

To determine concentrations of NMT and DMT, 10 µL of the 30% methanol resuspension (see ‘Enzyme assays’ section) was spiked with 10 µL of a mixture containing 100 nM d3-NMT, and d6-DMT (Toronto Research Chemicals) as deuterated internal standards to normalize for extraction efficiency and mass spectrometry ionization efficiency. Calibration solutions of NMT and DMT were prepared in 30% methanol, to create a calibration range of 0.625–125 nM for NMT, and 0.125–25 nM for DMT. Calibration standards were spiked with the internal standard solution as described above and injected prior to each day of analysis. 6-point calibration curves were determined based on the peak area ratio of the calibration standard to the internal standard by linear regression. All samples and standards were analyzed using a Phenomenex Kinetex C18 chromatography column (100 × 2.1 mm, 1.7 μm, 100 Å) on a Vanquish uHPLC (Thermo Fisher Scientific, Gemering, Germany) interfaced to a TSQ Quantum Ultra triple quadrupole mass spectrometer (Thermo Fisher Scientific, San Jose, CA). Mobile phase A was 10 mM ammonium formate with 0.15% (v/v) formic acid in water. Mobile phase B was acetonitrile. The gradient used was as follows: initial, 5% B; 0.01 min, 19% B; 0.68 min, 26% B, 1.05 min, 75% B; 1.8 min, 100% B; 2.2 min, 100% B; 2.3 min, 5% B; 3.0 min, 5% B at 600 μL/min. The sample injection volume was 7.5 µL, the autosampler was kept at ambient temperature, and the column was held at 30 °C in still air mode. NMT eluted at 0.92 min, and DMT at 0.96 min. Electrospray ionization was used in positive mode at 4 kV. The capillary temperature was 325 °C, the vaporizer temperature was 300 °C, the sheath gas pressure was 50, and the auxiliary gas pressure was 10. Ions were detected in tandem mass spectrometry mode. The scanning parameters for all 4 analytes were as follows. NMT: precursor ion m/z = 175, product ion m/z = 143.8, collision energy = 14, tube lens = 53. d3-NMT: precursor ion m/z = 178, product ion m/z = 143.8, collision energy = 14, tube lens = 65. DMT: precursor ion m/z = 189.1, product ion m/z = 144.1, collision energy = 19, tube lens = 49. d6-DMT: precursor ion m/z = 195.1, product ion m/z = 144.1, collision energy = 19, tube lens = 49. Automated peak integration was performed using XCalibur 3.0 MS software, and all peaks were visually inspected to ensure proper integration.

### Statistical analyses

Statistical analyses were conducted using GraphPad Prism version 9.4.1 (La Jolla, CA). For radiometric enzyme assays, all data exhibited a normal distribution confirmed via Shapiro–Wilk test of normality. Unpaired, parametric, two-tailed t-tests were used with Welch’s correction for all statistical comparisons. In the text, *p*-values, means ± standard deviations, means of differences, 95% confidence intervals (C.I.), and t values are reported. *α* = 0.05 for all tests.

## Supplementary Information

Supplementary Information 1.

Supplementary Information 2.

Supplementary Information 3.

Supplementary Information 4.

Supplementary Information 5.

Supplementary Information 6.

Supplementary Information 7.

Supplementary Information 8.

Supplementary Information 9.

Supplementary Information 10.

## Data Availability

Data and reagents will be available upon reasonable request from the corresponding author (J.B.).
