# Supplementary material for: Indolethylamine N-methyltransferase (INMT) is not essential for endogenous tryptamine-dependent methylation activity in rats
Source: Sci Rep. 2023 Jan 6;13:280. doi: 10.1038/s41598-023-27538-y (PMC9822953; doi:10.1038/s41598-023-27538-y)
Supplement: Supplementary file 1 — Supplementary Information 1. [file 41598_2023_27538_MOESM1_ESM.docx]

**Supplementary Materials**

**Indolethylamine *N*-Methyltransferase (INMT) is Not Essential for Endogenous Tryptamine-Dependent Methylation Activity in Rats**

Nicolas G. Glynos^#1,2^, Lily Carter^#1^, Soo Jung Lee^3,4^, Youngsoo Kim^5^, Robert T. Kennedy^5^, George A. Mashour^2,6,7^, Michael M. Wang^1,3,4,7^, Jimo Borjigin*^1,2,3,7^

1. Department of Molecular & Integrative Physiology, University of Michigan, Ann Arbor, MI, USA
2. Michigan Psychedelic Center, University of Michigan, Ann Arbor, MI, USA
3. Department of Neurology, University of Michigan, Ann Arbor, MI, USA
4. Veterans Affairs Ann Arbor Healthcare System, Ann Arbor, MI, USA
5. Department of Chemistry, University of Michigan, Ann Arbor, MI, USA
6. Department of Anesthesiology, University of Michigan, Ann Arbor, MI, USA
7. Neuroscience Graduate Program, University of Michigan, Ann Arbor, MI, USA

*Corresponding author ([borjigin@umich.edu](mailto:borjigin@umich.edu))

^#^These two authors contributed equally.

**Supplementary Figure S1**: Uncropped image of western blot showing the presence of INMT (green bands) in WT, but not KO rat lung tissues. Tubulin (red bands) was used as a loading control.

**Supplementary Figure S2:** Uncropped, unprocessed phosphor imaging scan of radiometric enzyme assay products with tryptamine as substrate using extracts from brain and lung tissues of WT and KO rats following separation on silica gel plates using a mobile phase of N-butanol:acetic acid:water (12:3:5). A standard mixture (Std) containing tryptamine, NMT and DMT were clearly separated using these chromatography methods (left most lane). The solvent front (top) and spotting line (bottom) were marked for each condition before phosphor imaging to allow for calculation of R_F_ values.

**

**Supplementary Figure S3:** Uncropped image of western blot showing expression of GST-INMT fusion proteins of rat, rabbit and human INMTs in E. coli. The control lane is showing expression of an empty vector GST, where INMT is absent.


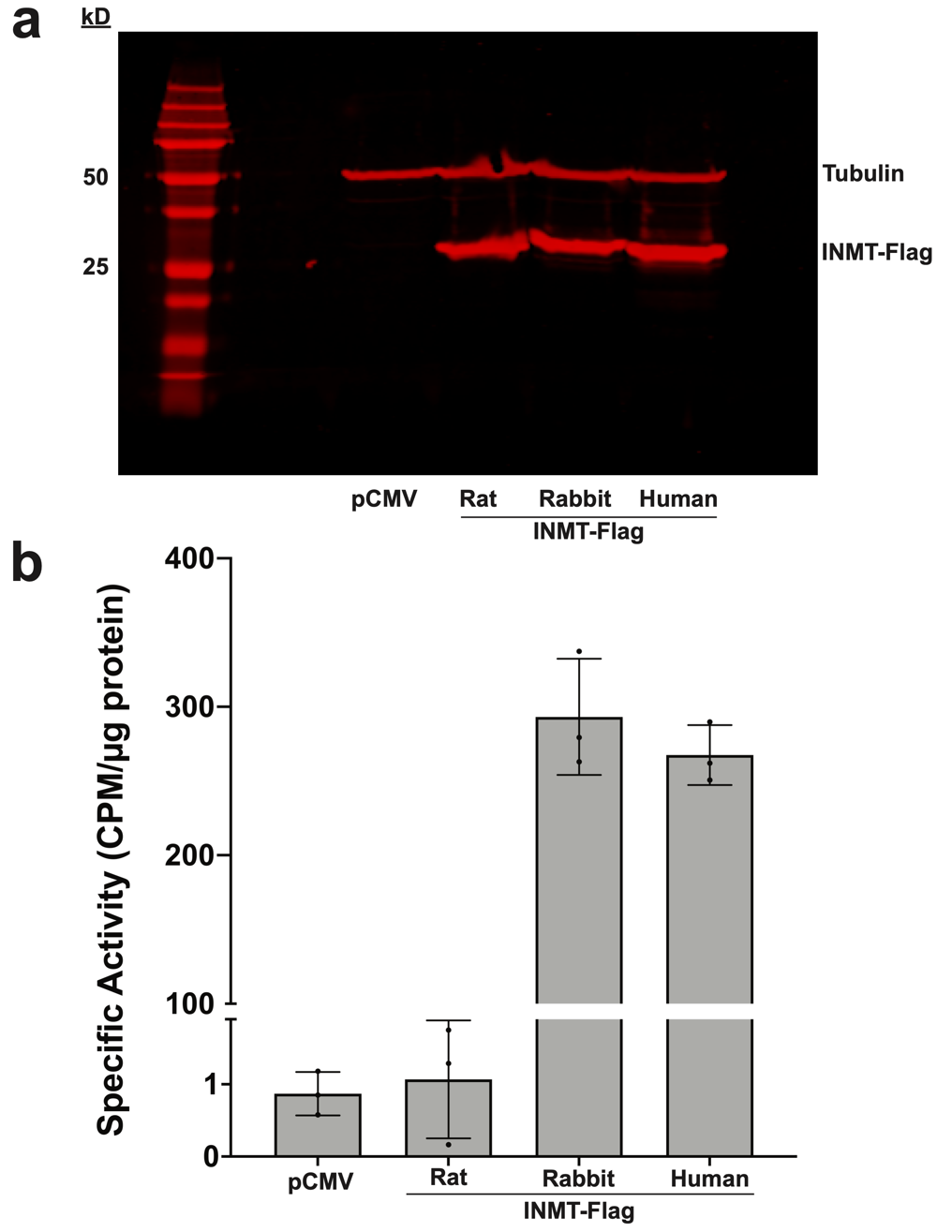


**Supplementary Figure S4:** **Rat INMT expressed in a mammalian cell line is not sufficient for tryptamine methylation.** (a) Uncropped image of western blot showing expression of Flag-INMT fusion proteins of rat, rabbit and human INMTs in human embryonic kidney (HEK) 293 cells. The control lane shows expression of an empty pCMV vector where INMT is absent. (b) Radiometric enzyme assays with tryptamine as substrate utilizing extracts from HEK293 cells expressing Flag-fusion recombinant rat, rabbit and human INMT proteins. Plots show the average of 3 individual experiments per condition with error bars indicating standard deviations. CPM = counts per minute.


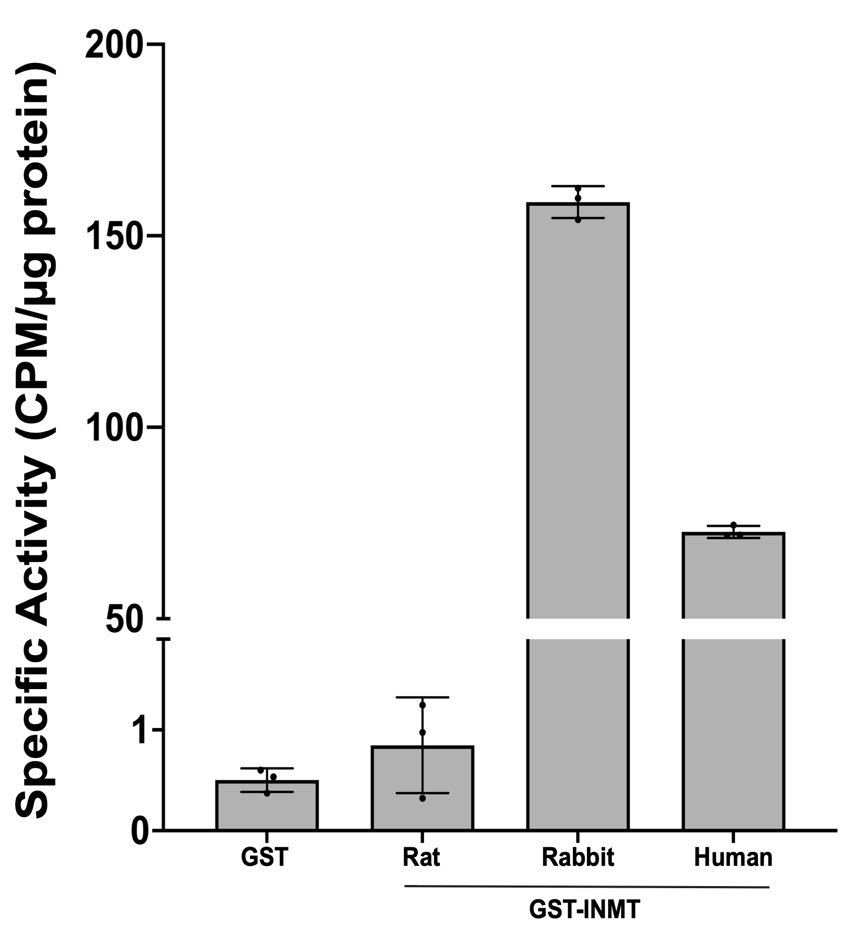


Supplementary Figure S5: **Rat INMT is not sufficient for NMT methylation.** Radiometric enzyme assays using NMT as substrate with extracts from E. coli expressing GST-fusion recombinant rat, rabbit, and human INMT proteins. Plots show the average of 3 individual experiments per condition with error bars indicating standard deviations. CPM = counts per minute.

**Supplementary Figure S6:** Uncropped, unprocessed phosphor imaging scan of radiometric enzyme assay products with tryptamine as substrate using recombinant GST-INMT fusion proteins following separation on silica gel plates using a mobile phase of N-butanol:acetic acid:water (12:3:5). A standard (Std) mixture containing tryptamine, NMT and DMT were clearly separated using these chromatography methods (left most lane and right most lane). The solvent front (top) and spotting line (bottom) were marked for each condition before phosphor imaging to allow for calculation of R_F_ values.

**Supplementary Figure S7:** **Rabbit and human INMT, but not rat INMT methylate NMT to produce DMT**. Phosphor imaging scan of radiometric enzyme assay products with NMT as substrate using recombinant GST-INMT fusion proteins following separation on silica gel plates using a mobile phase of N-butanol:acetic acid:water (12:3:5). A standard (Std) mixture containing tryptamine (R_F_ = 0.76), NMT (R_F_ = 0.67) and DMT (R_F_ = 0.55) were clearly separated using these chromatography methods (left most lane). Rabbit and human INMT produced spots isographic with DMT and had R_F_ values of 0.53 and 0.54, respectively. Uncropped, unprocessed phosphor imaging scan of the same graph is included as Supplementary Figure S8.

**Supplementary Figure S8:** Uncropped, unprocessed phosphor imaging scan of radiometric enzyme assay products with NMT as substrate using recombinant GST-INMT fusion proteins following separation on silica gel plates using a mobile phase of N-butanol:acetic acid:water (12:3:5). The solvent front (top) and spotting line (bottom) were marked for each condition before phosphor imaging to allow for calculation of R_F_ values.

| **Extracts** | **NMT** | **NMT (nM)** | **DMT (nM)** |
| --- | --- | --- | --- |
| GST | + | 812.61 ± 71.63 | NF |
| Rat GST-INMT | - | 0.32 ± 0.19 | NF |
| Rat GST-INMT | + | 937.56 ± 60.18 | NF |
| Rabbit GST-INMT | - | 0.30 ± 0.18 | NF |
| Rabbit GST-INMT | + | 927.77 ± 142.98 | 2.73 ± 0.61 |
| Human GST-INMT | - | 0.39 ± 0.23 | NF |
| Human GST-INMT | + | 882.82 ± 261.74 | 2.66 ± 0.28 |

**Supplementary Table S1**: uHPLC-MS/MS confirmation of NMT methylation activity of recombinant rat, rabbit, and human INMTs. Values shown are averages of triplicates for each condition ± standard deviation. NF = peak not found
