# Supplementary figures and images for: Indolethylamine N-methyltransferase (INMT) is not essential for endogenous tryptamine-dependent methylation activity in rats
Source: Sci Rep. 2023 Jan 6;13:280. doi: 10.1038/s41598-023-27538-y (PMC9822953; doi:10.1038/s41598-023-27538-y)

**kD**

50

25

**Tubulin**

**INMT**

**Rat  
INMT**

**WT**

**INMT-KO**

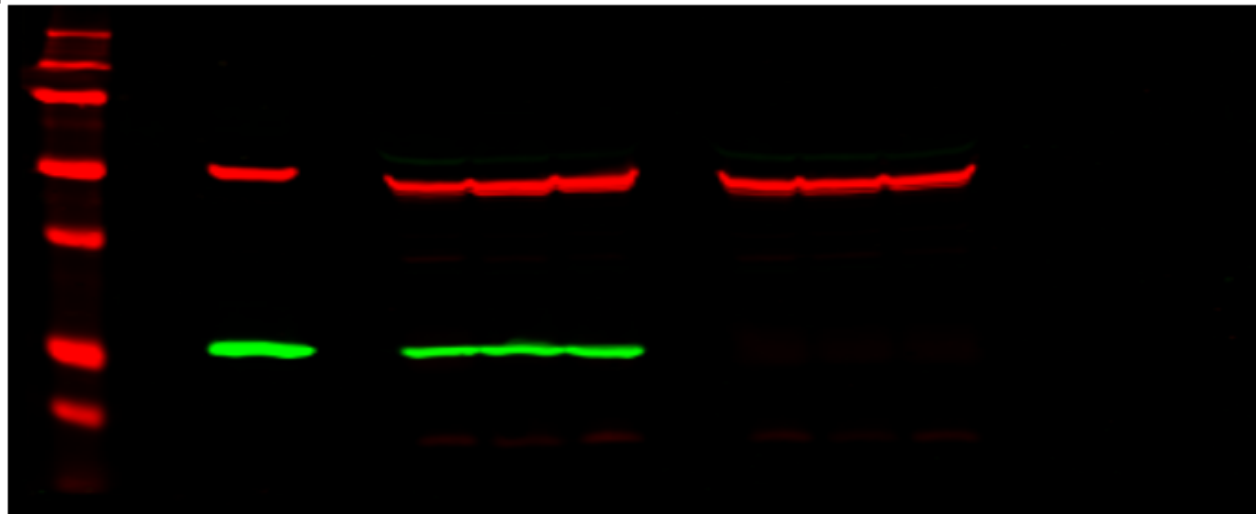

Supplement: Supplementary file 2 — Supplementary Information 2. [file 41598_2023_27538_MOESM2_ESM.pdf]

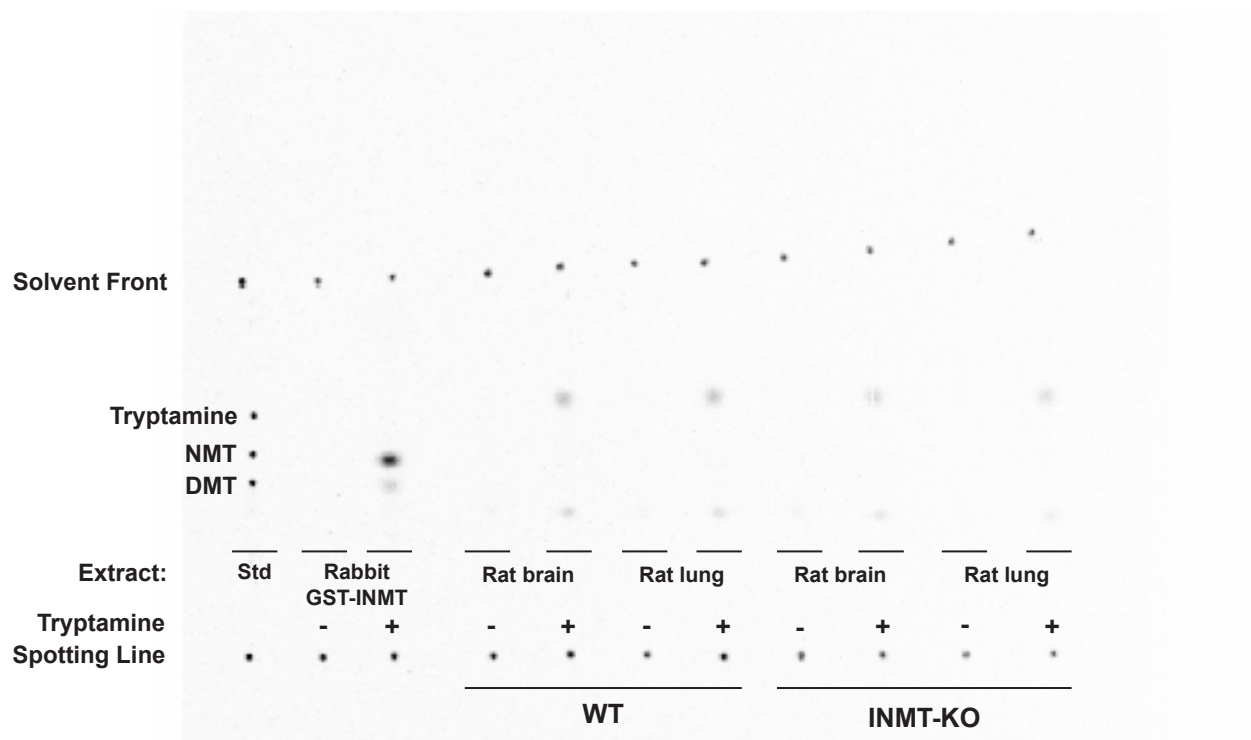

Supplement: Supplementary file 3 — Supplementary Information 3. [file 41598_2023_27538_MOESM3_ESM.pdf]

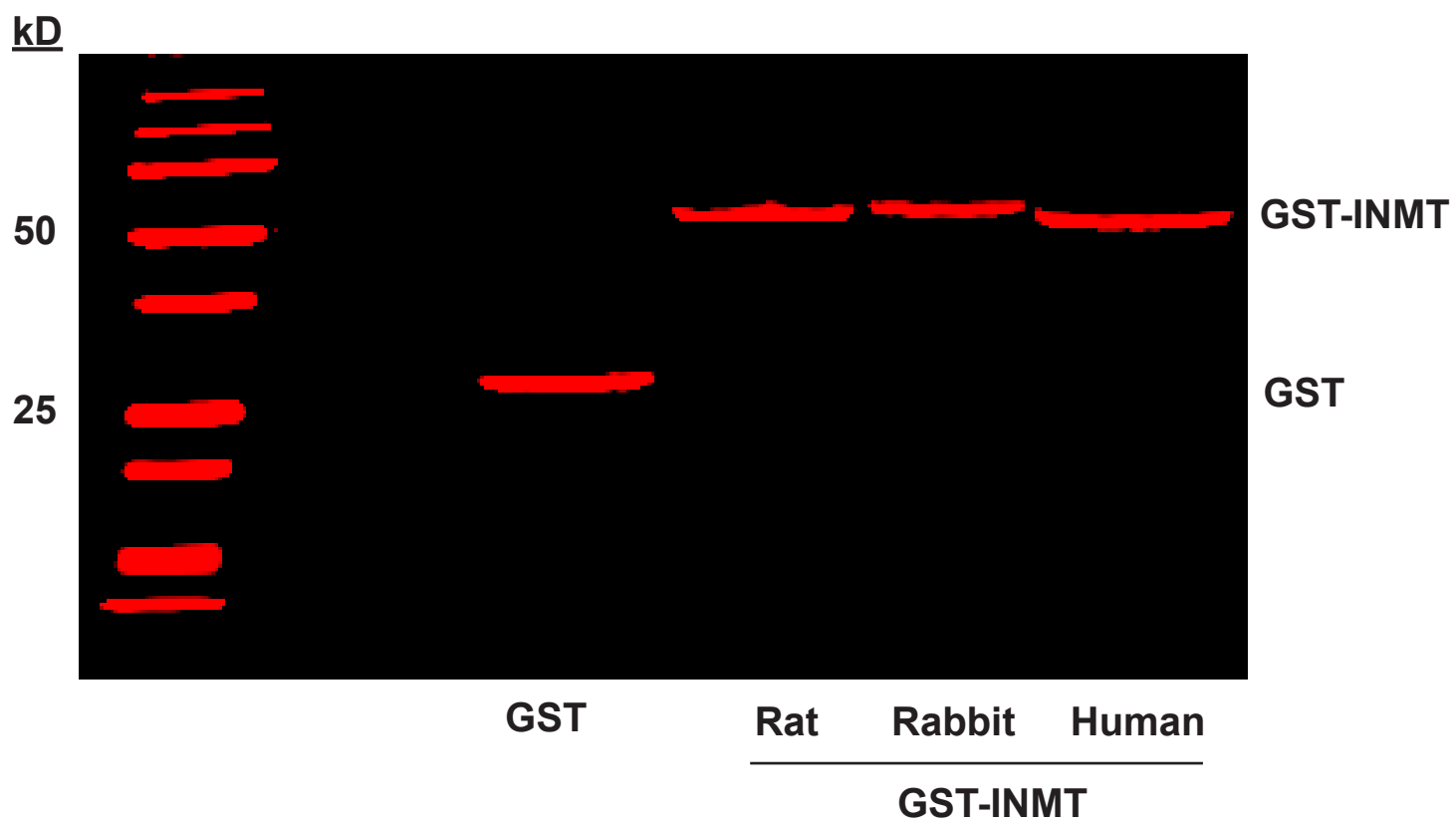

Supplement: Supplementary file 4 — Supplementary Information 4. [file 41598_2023_27538_MOESM4_ESM.pdf]

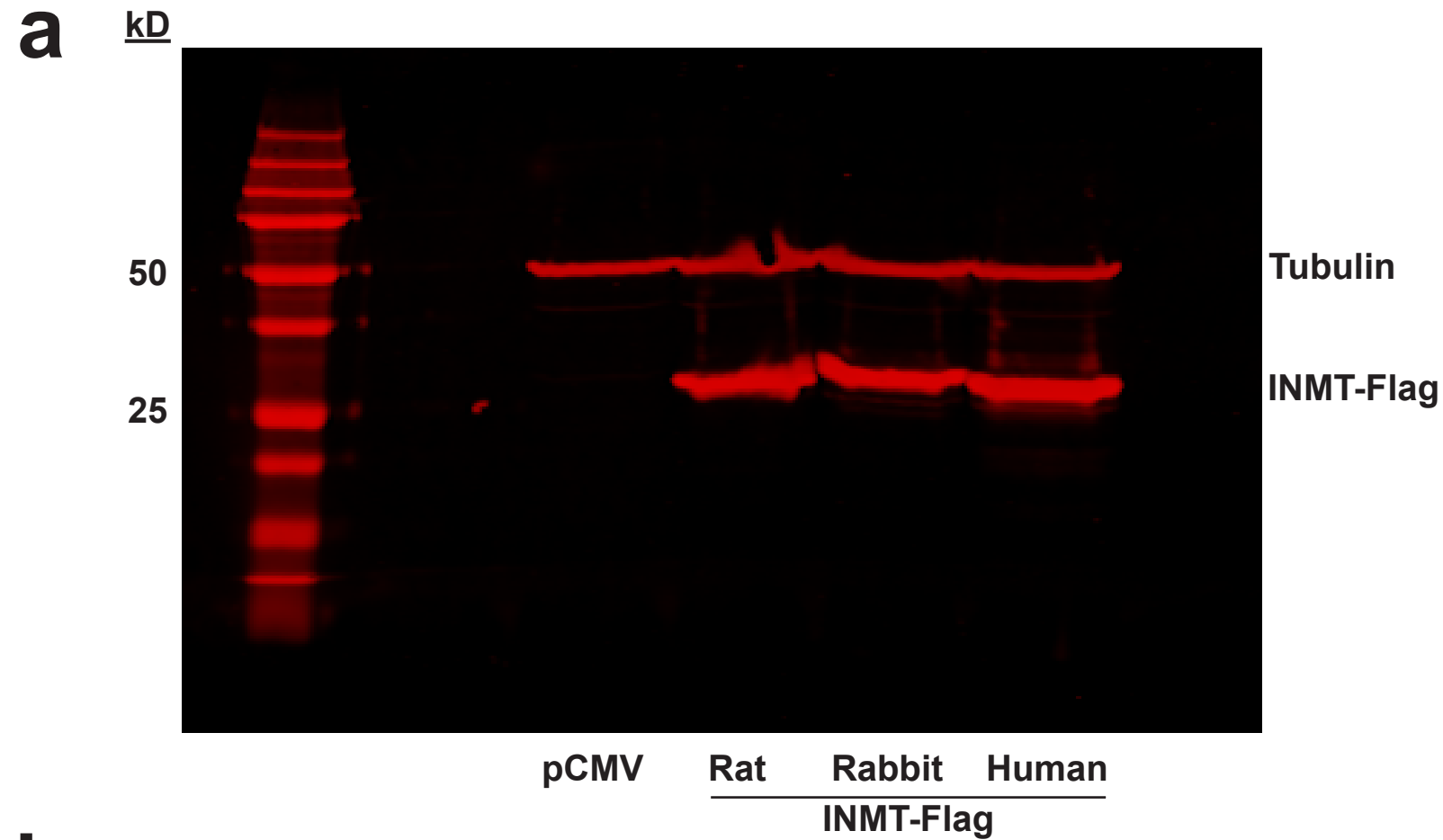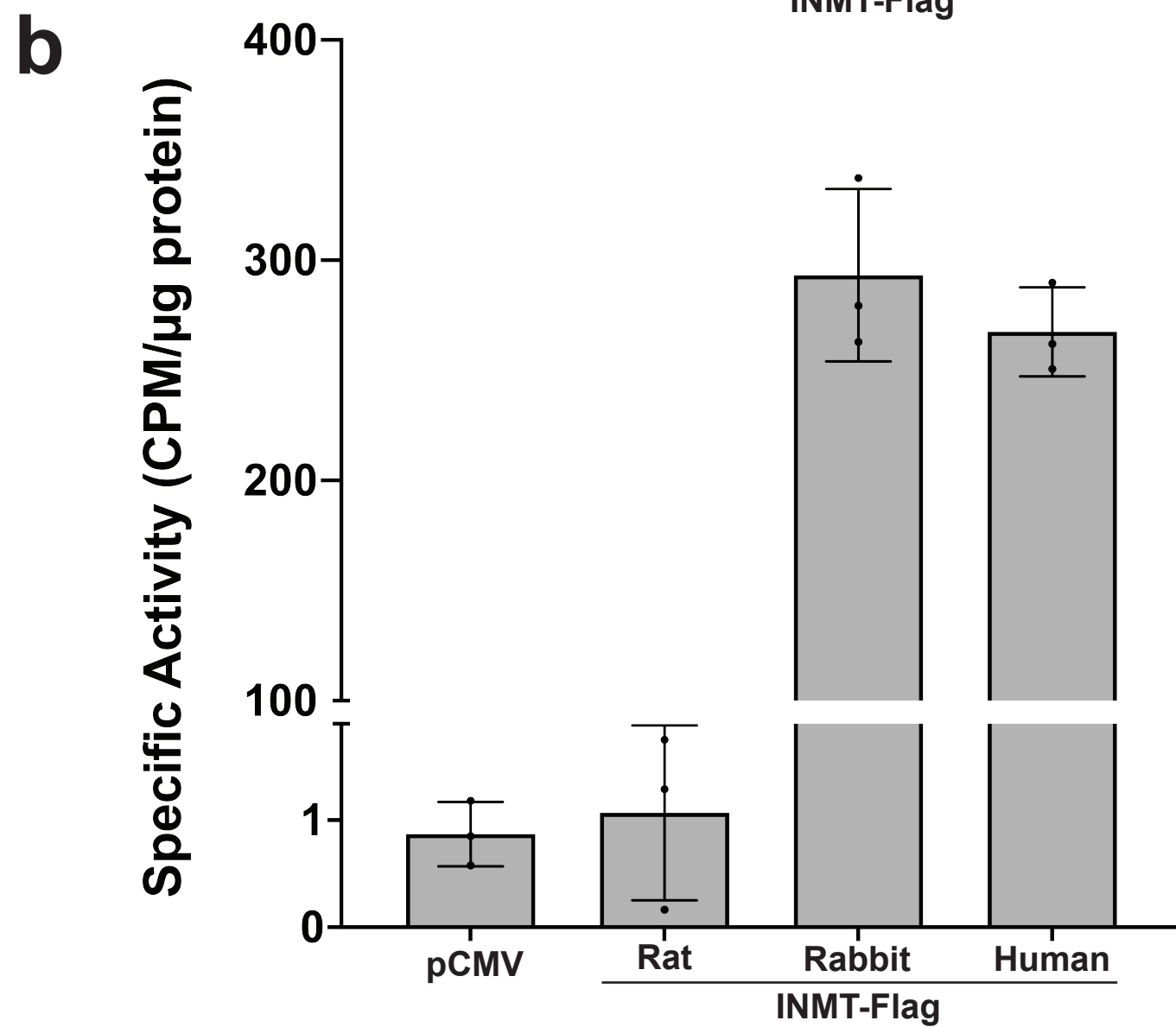

Supplement: Supplementary file 5 — Supplementary Information 5. [file 41598_2023_27538_MOESM5_ESM.pdf]

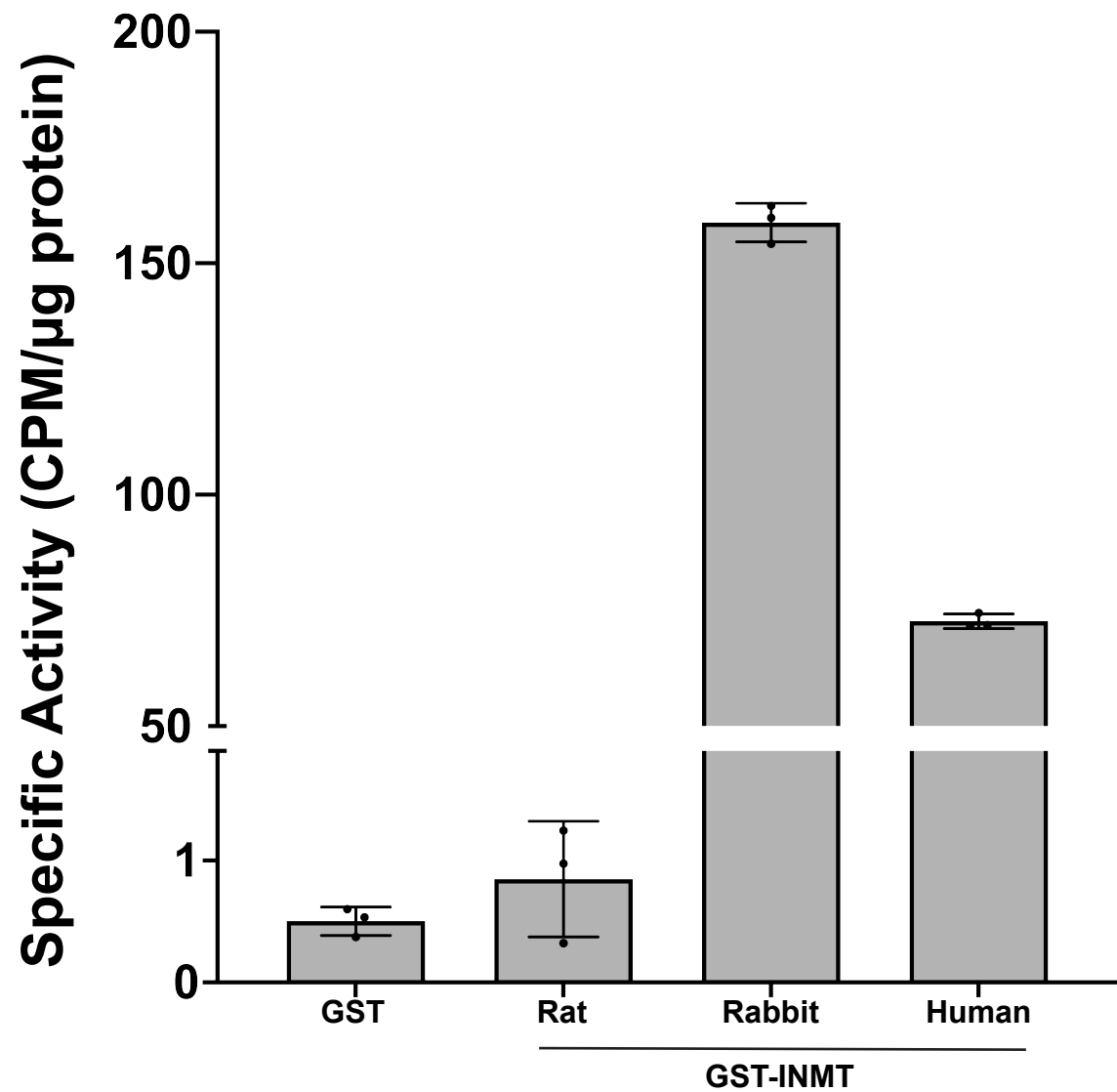

Supplement: Supplementary file 6 — Supplementary Information 6. [file 41598_2023_27538_MOESM6_ESM.pdf]

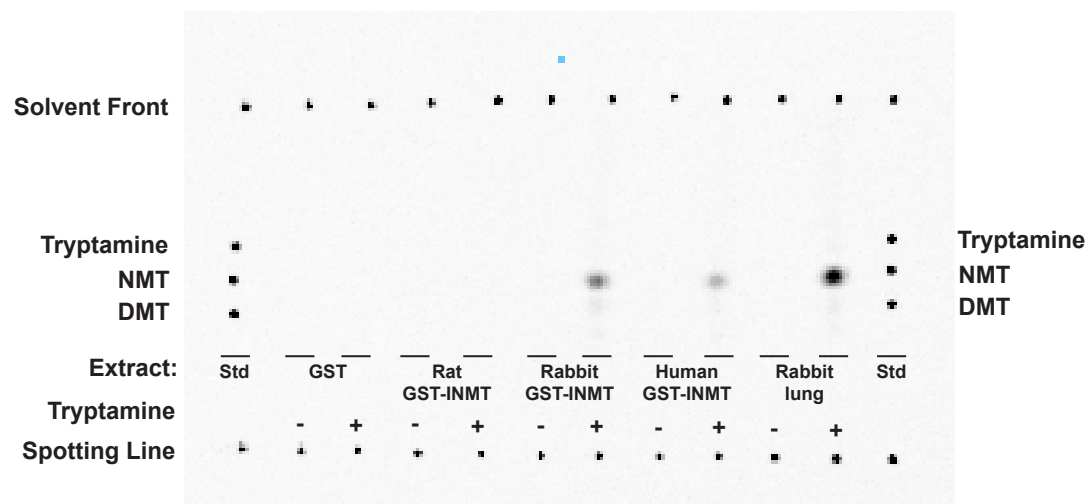

Supplement: Supplementary file 7 — Supplementary Information 7. [file 41598_2023_27538_MOESM7_ESM.pdf]

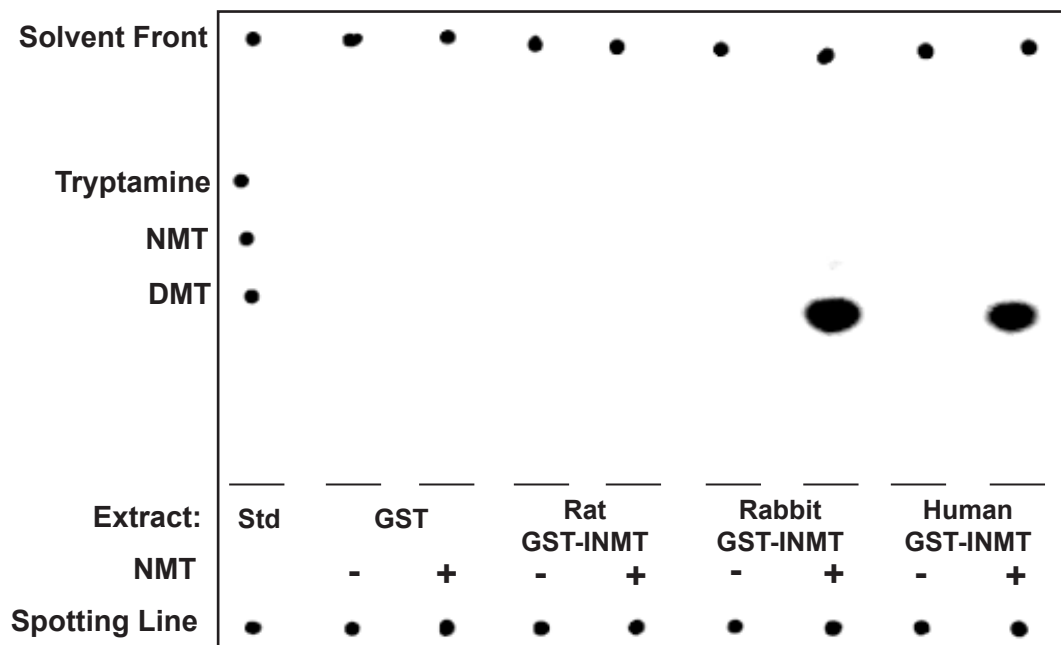

Supplement: Supplementary file 8 — Supplementary Information 8. [file 41598_2023_27538_MOESM8_ESM.pdf]

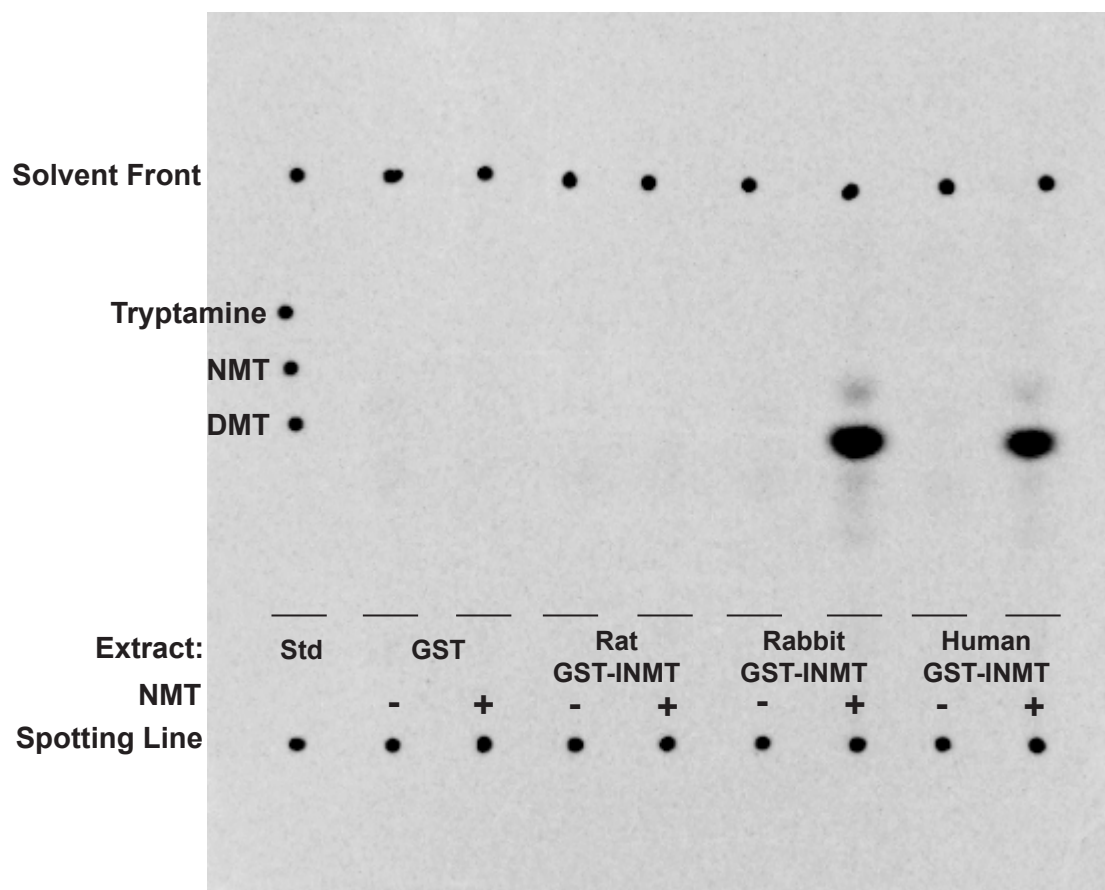

Supplement: Supplementary file 10 — Supplementary Information 10. [file 41598_2023_27538_MOESM10_ESM.pdf]
